# A new real-time PCR method to overcome significant quantitative inaccuracy due to slight amplification inhibition

**DOI:** 10.1186/1471-2105-9-326

**Published:** 2008-07-30

**Authors:** Michele Guescini, Davide Sisti, Marco BL Rocchi, Laura Stocchi, Vilberto Stocchi

**Affiliations:** 1 Istituto di Ricerca sull'Attività Motoria, Università degli Studi di Urbino "Carlo Bo", Via I Maggetti, 26/2 - 61029 Urbino, Italy; 2 Istituto di Chimica Biologica "G. Fornaini", Università degli Studi di Urbino "Carlo Bo", Via Saffi, 2 - 61029 Urbino, Italy; 3 Istituto di Biomatematica, Università degli Studi di Urbino "Carlo Bo", Campus Scientifico Sogesta, Loc Crocicchia, - 61029 Urbino, Italy

## Abstract

**Background:**

Real-time PCR analysis is a sensitive DNA quantification technique that has recently gained considerable attention in biotechnology, microbiology and molecular diagnostics. Although, the cycle-threshold (*Ct*) method is the present "gold standard", it is far from being a standard assay. Uniform reaction efficiency among samples is the most important assumption of this method. Nevertheless, some authors have reported that it may not be correct and a slight PCR efficiency decrease of about 4% could result in an error of up to 400% using the *Ct *method. This reaction efficiency decrease may be caused by inhibiting agents used during nucleic acid extraction or copurified from the biological sample.

We propose a new method (*Cy*_*0*_) that does not require the assumption of equal reaction efficiency between unknowns and standard curve.

**Results:**

The *Cy*_*0 *_method is based on the fit of Richards' equation to real-time PCR data by nonlinear regression in order to obtain the best fit estimators of reaction parameters. Subsequently, these parameters were used to calculate the *Cy*_*0 *_value that minimizes the dependence of its value on PCR kinetic.

The *Ct*, second derivative (*Cp*), sigmoidal curve fitting method (*SCF*) and *Cy*_*0 *_methods were compared using two criteria: precision and accuracy. Our results demonstrated that, in optimal amplification conditions, these four methods are equally precise and accurate. However, when PCR efficiency was slightly decreased, diluting amplification mix quantity or adding a biological inhibitor such as IgG, the *SCF*, *Ct *and *Cp *methods were markedly impaired while the *Cy*_*0 *_method gave significantly more accurate and precise results.

**Conclusion:**

Our results demonstrate that *Cy*_*0 *_represents a significant improvement over the standard methods for obtaining a reliable and precise nucleic acid quantification even in sub-optimal amplification conditions overcoming the underestimation caused by the presence of some PCR inhibitors.

## Background

In the last few years, the real-time polymerase chain reaction (PCR) has rapidly become the most widely used technique in modern molecular biology [1-4]. This technique relies on fluorescence-based detection of amplicon DNA and allows the kinetics of PCR amplification to be monitored in real time, making it possible to quantify nucleic acids with extraordinary ease and precision. With a large dynamic range (7–8 magnitudes) and a high degree of sensitivity (5–10 molecules), the real-time PCR addresses the evident requirement for quantitative data analysis in molecular medicine, biotechnology, microbiology and diagnostics [5,6].

Although, the real-time PCR analysis has gained considerable attention in many fields of molecular biology, it is far from being a standard assay. One of the problems associated with this assay, which has a direct impact on its reliability, is inconsistent data analysis. At the present, real-time PCR analysis is highly subjective and, if carried out inappropriately, confuses the actual results [7]. Many different options for data processing are currently available. The basic choice in real time PCR calculations is between absolute quantification, based on standard curve, and relative quantification, based on PCR efficiency calculation. Using the software currently available, analysis of real-time PCR data is generally based on the "cycle-threshold" method. The cycle-threshold is defined as the fractional cycle number in the log-linear region of PCR amplification in which the reaction reaches fixed amounts of amplicon DNA. There are two methods for determining the cycle-threshold value; one method, namely fit point, is performed by drawing a line parallel to the x-axis of the real-time fluorescence intensity curve (*Ct*) [8]. The second, namely second derivative, calculates the fractional cycle where the second derivative of the real-time fluorescence intensity curve reaches the maximum value (*Cp*) [9]. Standard curve method requires generating serial dilutions of a given sample and performing multiple PCR reactions on each dilution [10,11], the threshold-cycle values are then plotted versus the log of the dilution and a linear regression is performed from which the mean efficiency can be derived. This approach is only valid if the threshold-cycle values are measured from the exponential phase of the PCR reaction and if the efficiency is identical between amplifications. Furthermore, this efficiency is assumed to be the same for all the standard dilutions, but some authors have reported that this assumption may be questionable [12].

It is well-recognized that template quality is one of the most important determinants of real-time PCR reliability and reproducibility [13], and numerous authors have shown the significant reduction in the sensitivity and kinetics of real-time PCR assays caused by inhibitory components frequently found in biological samples [14-17]. The inhibiting agents may be reagents used during nucleic acid extraction or copurified components from the biological sample such as bile salts, urea, haeme, heparin, and immunoglobulin G. Inhibitors can generate strongly inaccurate quantitative results; while, a high degree of inhibition may even create false-negative results.

The *Ct *method is the most widely used method even though its calculation is user-dependent. The *Ct *method is quite stable and straightforward but the accuracy of estimates is strongly impaired if efficiency is not equal in all reactions. Indeed, uniform reaction efficiency is the most important assumption of the *Ct *method.

An alternative approach, proposed by Liu and Saint [18], assumes a dynamic change in efficiency fitting PCR amplification with a sigmoid function (Sigmoidal curve fitting method, *SCF*). One of the advantages of this regression analysis is that it allows us to estimate the initial template amount directly from the non-linear regression, eliminating the need for a standard curve. These pioneering works showed that it was possible to obtain absolute quantification from real-time fluorescence curve shape. However, recent reports have demonstrated that, in an optimized assay, the *Ct *method remains the gold standard due to the inherent errors of the multiple estimates used in non-linear regression [19,20].

We propose, in this report, a modified standard curve-based method (named *Cy*_*0*_) that does not require the assumption of uniform reaction efficiency between standards and unknown and does not involve any choice of threshold level by the user.

The aim of this work was also to compare the accuracy and precision of the *SCF*, *Ct*, *Cp *and *Cy*_*0 *_methods in presence of varying PCR kinetics. Our results clearly show that the proposed data processing procedure can effectively be applied in the quantification of samples characterized by slight amplification inhibition obtaining reliable and precise results.

## Methods

### Experimental design

The absolute quantification method relies on the comparison of distinct samples, such as the comparison of a biological sample with a standard curve of known initial concentration [21]. We wondered how accuracy and precision change when a standard curve is compared with unknown samples characterized by different efficiencies. A natural way of studying the effect of efficiency differences among samples on quantification would be to compare the amounts of a quantified gene.

A slight amplification inhibition in the quantitative real-time PCR experiments was obtained by using two systems: decreasing the amplification mix used in the reaction and adding varying amounts of IgG, a known PCR inhibitor.

For the first system, we amplified the MT-ND1 gene by real-time PCR in reactions having the same initial amount of DNA but different amounts of SYBR Green I Master mix. A standard curve was performed over a wide range of input DNA (3.14 × 10^7^–3.14 × 10^1^) in the presence of optimal amplification conditions (100% amplification mix), while the unknowns were run in the presence of the same starting DNA amounts but with amplification mix quantities ranging from 60% to 100%. This produced different reaction kinetics, mimicking the amplification inhibition that often occurs in biological samples [17,22].

Furthermore, quantitative real-time PCR quantifications were performed in the presence of an optimal amplification reaction mix added with serial dilutions of IgG (0.0625 – 2 μg/ml) thus acting as the inhibitory agent [23].

The reaction efficiency obtained was estimated by the LinReg method [24]. This approach identifies the exponential phase of the reaction by plotting the fluorescence on a log scale. A linear regression is then performed leading to the estimation of the efficiency of each PCR reaction.

### Quantitative Real-Time PCR

The DNA standard consisted of a pGEM-T (Promega) plasmid containing a 104 bp fragment of the mitochondrial gene NADH dehydrogenase 1 (MT-ND1) as insert. This DNA fragment was produced by the ND1/ND2 primer pair (forward ND1: 5'-ACGCCATAAAACTCTTCACCAAAG-3' and reverse ND2: 5'-TAGTAGAAGAGCGATGGTGAGAGCTA-3'). This plasmid was purified using the Plasmid Midi Kit (Qiagen) according to the manufacturer's instructions. The final concentration of the standard plasmid was estimated spectophotometrically by averaging three replicate A_260 _absorbance determinations.

Real time PCR amplifications were conducted using LightCycler^® ^480 SYBR Green I Master (Roche) according to the manufacturer's instructions, with 500 nM primers and a variable amount of DNA standard in a 20 μl final reaction volume. Thermocycling was conducted using a LightCycler^® ^480 (Roche) initiated by a 10 min incubation at 95°C, followed by 40 cycles (95°C for 5 s; 60°C for 5 s; 72°C for 20 s) with a single fluorescent reading taken at the end of each cycle. Each reaction combination, namely starting DNA and amplification mix percentage, was conducted in triplicate and repeated in four separate amplification runs. All the runs were completed with a melt curve analysis to confirm the specificity of amplification and lack of primer dimers. *Ct *(fit point method) and *Cp *(second derivative method) values were determined by the LightCycler^® ^480 software version 1.2 and exported into an MS Excel data sheet (Microsoft) for analysis after background subtraction (available as Additional file 1). For *Ct *(fit point method) evaluation a fluorescence threshold manually set to 0.5 was used for all runs.

### Description of the *SCF *method

Fluorescence readings were used to fit the following 4-parameter sigmoid function using nonlinear regression analysis:

(1) $$F_{x}=\frac{F_{\max}}{1+e^{\left(-\frac{1}{b}\left(x-c\right)\right)}}+F_{b}$$

where *x *is the cycle number, *F*_*x *_is the reaction fluorescence at cycle *x*, *F*_*max *_is the maximal reaction fluorescence, *c *is the fractional cycle at which reaction fluorescence reaches half of *F*_*max*_, *b *is related to the slope of the curve and *F*_*b *_is the background reaction fluorescence. *F*_*max *_quantifies the maximal fluorescence read by the instrument and does not necessarily indicate the amount of DNA molecules present at the end of the reaction. The fact that *F*_*max *_does not necessarily represent the final amount of DNA might be due to un-saturating dye concentration or to fluorescence quenching by inhibitors. For each run a nonlinear regression analysis was performed and these four parameters were evaluated. A simple derivative of Eq. 1 allowed us to estimate *F*_*0*_, when *x *= 0:

(2) $$F_{0}=\frac{F_{\max}}{1+e^{\left(\frac{c}{b}\right)}}$$

where *F*_*0 *_represents the initial target quantity expressed in fluorescence units. Conversion of *F*_*0 *_to the number of target molecules was obtained by a calibration curve in which the log input DNA was related to the log of *F*_*0 *_[18]. Subsequently, this equation was used for quantification with log transformation of fluorescence data to increase goodness-of-fit as described in Goll *et al*. 2006 [19].

### Description of the *Cy*_*0 *_method

**Figure 1:**
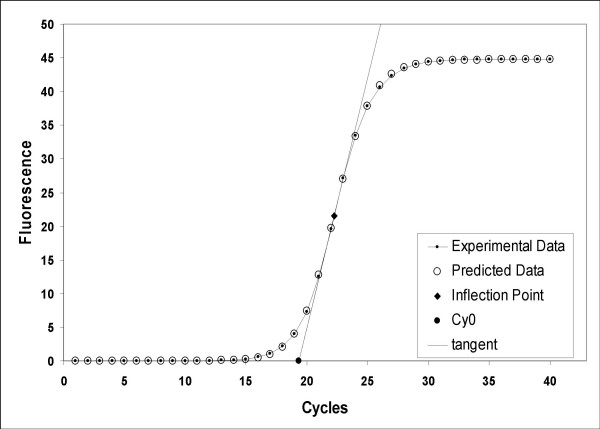
**Example of modelling PCR amplification with a 5-parameter Richards function**. Effectiveness of this model is illustrated by the predicted values generated by Eq. 3 (open circles) that agree with the observed fluorescence (dot and line). Curve-fitting of experimentally derived fluorescence dataset to Eq. 3 generates values for the kinetic parameters from which the inflection point (solid black rhombus) and the slope of the curve can be derived. The quantitative entity *Cy*_*0 *_(solid black dot), used in the proposed method, shows the cross point between the x-axis and the tangent crossing the inflection point of real-time PCR fluorescence curve.

The *Cy*_*0 *_value is the intersection point between the abscissa axis and tangent of the inflection point of the Richards curve obtained by the non-linear regression of raw data (Fig. 1).

The *Cy*_*0 *_method was performed by nonlinear regression fitting of the Richards function [25], an extension of logistic growth curve, in order to fit fluorescence readings to the 5-parameter Richards function:

(3) $$F_{x}=\frac{F_{\max}}{{\left[1+e^{\left(-\frac{1}{b}\left(x-c\right)\right)}\right]}^{d}}+F_{b}$$

where *x *is the cycle number, *F*_*x *_is the reaction fluorescence at cycle *x*, *F*_*max *_is the maximal reaction fluorescence, *x *is the fractional cycle of the turning point of the curve, *d *represents the Richards coefficient, and *F*_*b *_is the background reaction fluorescence. The inflection point coordinate (*Flex*) was calculated as follows (Additional file 2):

(4) $$Flex=\left[c+b\ln d;F_{\max}{\left(\frac{d}{d+1}\right)}^{d}+F_{b}\right]$$

and the tangent slope (*m*) was estimated as:

(5) $$m=\frac{F_{\max}}{b}{\left(\frac{d}{d+1}\right)}^{d+1}$$

When *d *= 1, the Richards equation becomes the logistic equation shown above. The five parameters that characterized each run were used to calculate the *Cy*_*0 *_value by the following equation:

(6) $$C_{y0}=c+b\ln d-b\left(\frac{d+1}{d}\right)\left[1-\frac{F_{b}}{F_{\max}}{\left(\frac{d+1}{d}\right)}^{d}\right]$$

Although the *Cy*_*0 *_is a single quantitative entity, as is the *Ct *or *Cp *for threshold methodologies, it accounts for the reaction kinetic because it is calculated on the basis of the slope of the inflection point of fluorescence data.

### Statistical data analysis

Nonlinear regressions (for 4-parameter sigmoid and 5-parameter Richards functions) were performed determining unweighted least squares estimates of parameters using the Levenberg-Marquardt method. Accuracy was calculated using the following equation:

$RE_{\left(n_{Dna},{\%}_{mix}\right)}=\sum_{i=1}^{n}\left(\frac{x_{i_{obs}\left(n_{Dna},{\%}_{mix}\right)}}{x_{i_{\exp}\left(n_{Dna},{\%}_{mix}\right)}}-1\right)$, where $RE_{\left(n_{Dna},{\%}_{mix}\right)}$ was the relative error, while $x_{i_{obs}\left(n_{Dna},{\%}_{mix}\right)}$ and $x_{i_{\exp}\left(n_{Dna},{\%}_{mix}\right)}$ were the estimated and the true number of DNA molecules for each combination of input DNA (*n*_*Dna*_) and amplification mix percentage (*%*_*mix*_) used in the PCR. Precision was calculated as:

$CV_{\left(n_{Dna},{\%}_{mix}\right)}=\frac{s_{{\bar{x}}_{obs\left(n_{Dna},{\%}_{mix}\right)}}}{{\bar{x}}_{obs\left(n_{Dna},{\%}_{mix}\right)}}$, where $CV_{\left(n_{Dna},{\%}_{mix}\right)}$ was the coefficient of variation, ${\bar{x}}_{obs\left(n_{Dna},{\%}_{mix}\right)}$ and $s_{{\bar{x}}_{obs\left(n_{Dna},{\%}_{mix}\right)}}$ were the mean and the standard deviation for each combination of n_Dna _and %_mix_. In order to verify that the Richards curves, obtained by nonlinear regression of fluorescence data, were not significantly different from the sigmoidal curves, the values of *d *parameter were compared to the expected value *d *= 1, using *t *test for one sample. For each combination of *n*_*Dna*_, *%*_*mix*_, the *t *values were calculated as follow:

$t_{\left(n_{Dna},{\%}_{mix}\right)}=\frac{{\bar{d}}_{\left(n_{Dna},{\%}_{mix}\right)}-1}{SE_{d_{\left(n_{Dna},{\%}_{mix}\right)}}}$, where ${\bar{d}}_{\left(n_{Dna},{\%}_{mix}\right)}$ and $SE_{d_{\left(n_{Dna},{\%}_{mix}\right)}}$ were the mean and the standard error of *d *values for each combination of *n*_*Dna *_and *%*_*mix*_, with p(*t*) < 0.05 for significance level. $RE_{\left(n_{Dna},{\%}_{mix}\right)}$ values were reported using 3-d scatterplot graphic, a complete second order polinomial regression function was shown to estimate the trend of accuracy values. $CV_{\left(n_{Dna},{\%}_{mix}\right)}$ where also reported using 3-d contour plots using third-order polynomials spline fitting. All elaborations and graphics were obtained using Excel (Microsoft), Statistica (Statsoft) and Sigmaplot 10 (Systat Software Inc.).

## Results

### Experimental system 1: reduction of amplification mix percentage

**Figure 2:**
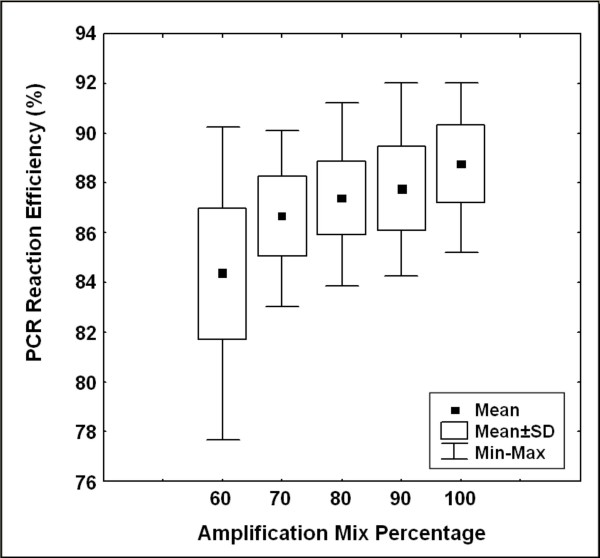
**Estimation of PCR efficiency using LinReg method**. Efficiency values were determined from 420 independent reactions using a combination of 3.14 × 10^7^–3.14 × 10^1 ^DNA molecules as starting template and amplification mix quantities ranging from 60% to 100%. The graph shows the distribution of PCR efficiencies in relation to the percentage of amplification mix used in the reaction. The solid black squares (▪) represent the mean of each distribution.

With our experimental set up, the mean PCR reaction efficiency was 88% under optimal amplification conditions and slightly decreased in the presence of smaller amplification mix up to 84%. Moreover, for decreasing amplification mix amounts, the PCR reaction efficiencies showed higher dispersion levels than optimal conditions leading to increasing quantitative errors (Variation Interval, VI_100% _= 92%–85% and VI_60% _= 90%–77%; Fig. 2). Subsequently, the fluorescence data obtained in these reactions were used to calculate the initial DNA amount using four different procedures: *SCF*, *Ct*, *Cp *and *Cy*_*0*_.

### Precision and accuracy of the *SCF *method

Previous studies have shown that the *SCF *approach can lead to quantification without prior knowledge of amplification efficiency [18,19,26]; therefore, we evaluated the performance of this method on our data set. To assess the effect of unequal efficiencies on accuracy, the calculated input DNA, expressed as molecular number, was compared to the expected value obtaining the relative error (RE). The precision was further evaluated measuring the variation coefficient (CV%) of the estimated initial DNA in the presence of different PCR efficiencies and input DNA.

In our experimental design, the *SCF *method showed a very poor precision (mean CV% = 594.74%) and low accuracy (mean RE = -5.05). The impact of amplification efficiency decline on accuracy was very strong resulting in an underestimate of samples of up to 500% (Additional file 3). The log transformation of fluorescence data before sigmoidal fitting significantly reduced the CV% and RE to 66.12% and -0.20, respectively; however, the overall bias remained the same [19]. Finally, we also tested an improved *SCF *approach based on a previous study by Rutledge 2004 [26] without obtaining significant amelioration (Additional file 4).

### The *Cy*_*0 *_method

**Figure 3:**
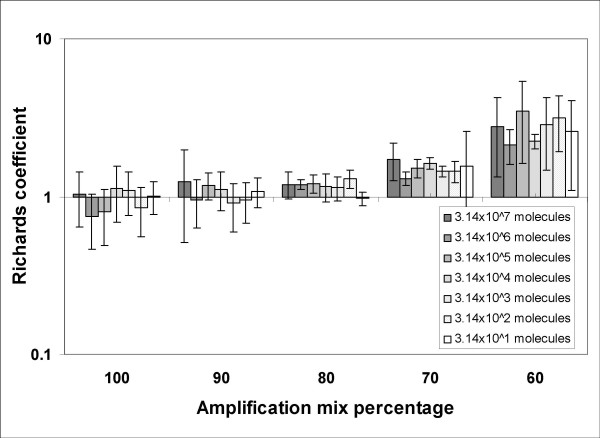
**Distribution of Richards coefficients (*d*) estimated from PCR fluorescence curves using Eq. 3 in nonlinear fitting procedure**. Richards coefficient values were determined from 420 independent PCR reactions. The data have been reported in Log_10 _scale, and represented as mean and standard deviation.

The *SCF *model assumes that the fluorescence signal is proportional to the amount of product, which is often the case for SYBR-Green I real-time PCR performed with saturing concentrations of dye. In such conditions, centrally symmetric amplification curves are expected. However, in our experience, we found several non-symmetric amplification curves shown to have good amplification efficiency using standard curve analysis (Additional file 1 and 3). In order to find a suitable mathematical representation of the complete PCR kinetic curve we compared the standard error of estimate obtained by several equations that generate S-shaped curves (Tab. 1). As shown in Figure 1, these results demonstrated that real-time PCR readouts can be effectively modelled using the 5-parameter Richards function (Eq. 3). The Richards equation is an extension of the sigmoidal growth curve; specifically, when *d *coefficient is equal to 1, the sigmoidal and Richards curves are the same. Hence, we analysed the variation of the *d *coefficient in the presence of different input DNA and PCR efficiencies. Figure 3 shows that the *d *value is close to 1 at amplification mix percentages ranging from 100% to 90% while at lower amplification mix contents, where PCR efficiency decreases, the *d *coefficient was significantly higher than 1 regardless of the starting DNA content (Fig. 3; Tab. 2). These data demonstrate that sigmoidal fitting represents a good approximation of real-time PCR kinetic only in the presence of optimal amplification conditions while the Richards curve is more suited when PCR is inhibited. Since the Richards growth equation includes sigmoidal amplification curves, when *d *= 1, this nonlinear fitting was used in our method.

**Table 1:** Comparison of five S-shaped models to fit the PCR curve: Sigmoid, Richards, Gompertz, Hill and Chapman.

***Name***	***Equation***	***Estimated Parameters***					***R***^2^	***Adj R***^2^	***Standard Error of Estimate***
					
		***F***_*max*_	***b***	***c***	***F***_*b*_	***d***			
**Sigmoid**	*f *= *F*_*b*_+*F*_*max*_/(1+exp(-(*x*-*c*)/*b*))	45.11	1.49	22.37	-0.03		1	1	0.1354
**Richards**	*f *= *F*_*b*_+(*F*_*max*_/(1+exp(-(1/*b*)*(*x*-*c*)))^*d*)	45.11	1.58	21.95	0.02	1.20	1	1	**0.0926**
**Gompertz**	*f *= *F*_*b*_+*F*_max _*exp(-exp(-(*x*-*c*)/*b*))	45.19	2.15	21.45		0.29	0.9992	0.9992	0.6006
**Hill**	*f *= *F*_*b*_+*F*_max _*x^*b*/(*d*^*b*+*x*^*b*)	45.18	14.95		0.08	22.34	1	1	0.1351
Chapman	*f *= *F*_*b*_+*F*_max _*(1-exp(-b*x))^*d*	45.19	0.46		0.29	20615	0.9992	0.9992	0.6006

In this table, *f *is the fluorescence at cycle *x*; *F*_*max *_represents the maximum fluorescence value; *F*_*b *_is the background reaction fluorescence; *b*, *c *and *d *determine the shape of each curve. For each model the determination coefficient (R^2^), the adjusted determination coefficient (Adj R^2^) and the standard error of estimate have been calculated.

**Table 2:** *t *statistic values obtained for all variable combinations.

	**Amplification mix percentage**				
	
**Log**_10_**input DNA**	**100%**	**90%**	**80%**	**70%**	**60%**
**7.5**	0.28348	1.15431	2.9303*	5.43493**	4.26067**
**6.5**	-3.0233*	-0.5329	7.8552**	8.68609**	7.28178**
**5.5**	-2.2195*	2.70419*	4.7185**	8.61406**	4.60465**
**4.5**	0.97856	1.32162	2.34*	16.5192**	17.5903**
**3.5**	1.00647	-1.038	2.3307*	13.2572**	4.65683**
**2.5**	-1.731	-0.5995	5.8385**	6.90378**	6.13465**
**1.5**	0.14417	1.25452	-0.898	1.87978	3.69668**

When *t *< 0 the Richards coefficient is lower than 1, while for *t *> 0 the Richards coefficient is higher than 1. Significance levels: * 0.05 <*p *< 0.01; ** *p *< 0.01

**Figure 4:**
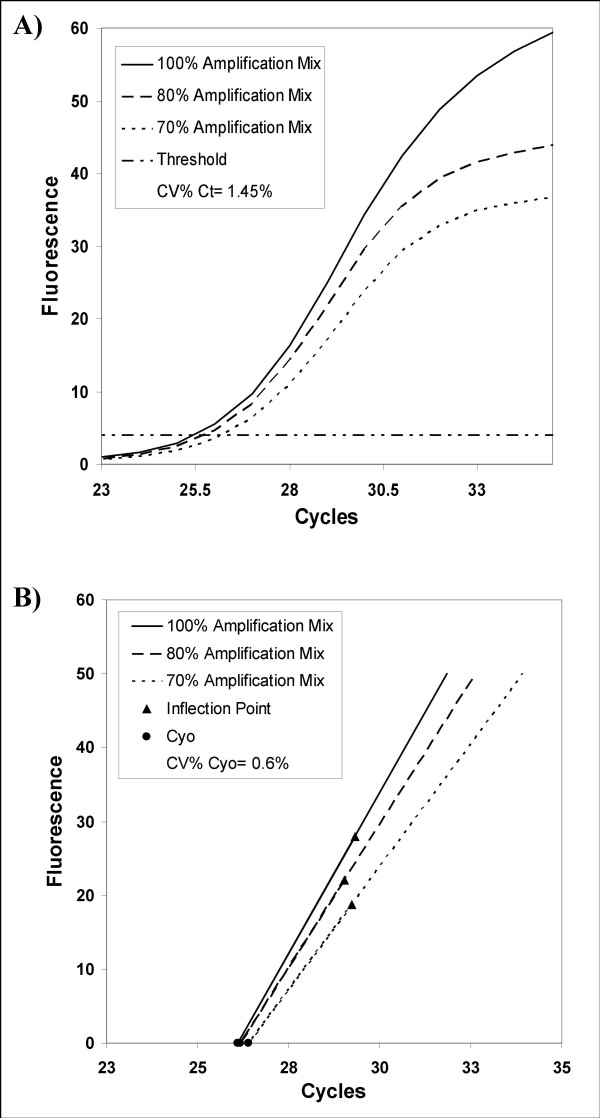
**Plot of fluorescence observations versus cycle number obtained from the same starting DNA but in presence of decreasing amounts of amplification mix**. This slight PCR inhibition produces curves which are less steep than controls and shifted towards the right. When analysed by the threshold method, these curves showed higher *Ct *values with a CV% of 1.45% (A). An example of *Cy*_*0 *_procedure has been reported for the same data set (B). In this method, the amplification reactions are described by the tangent crossing the inflection point of fluorescence curves. As shown in this figure, the straight-lines originating from PCRs, characterized by slightly different PCR efficiency and the same starting amounts, tend to cross into a common point near the x-axis leading to small variations in the *Cy*_*0 *_values (CV% = 0.6%).

Despite the good fitting obtained by the Richards equation, the application of kinetic parameters to estimate *F*_*0 *_values showed a very low degree of precision and accuracy (Additional file 3). In an attempt to increase the reproducibility of outcomes a log transformation of fluorescence data was performed, however no satisfactory results were obtained (Additional file 3). To overcome these problems, we formulated an alternative method for starting DNA estimation that defines a new quantitative entity, *Cy*_*0*_. *Cy*_*0 *_can be considered similar to *Ct *or *Cp *but the main advantage of the *Cy*_*0 *_method is that it takes into account the kinetic parameters of amplification curve. This new method is based on the fit of Eq. 3 to real-time PCR data by nonlinear regression in order to obtain the best fit estimators of reaction parameters. In addition, these parameters were used to calculate the *Cy*_*0 *_value using Eq. 6. From a mathematical standpoint, the *Cy*_*0 *_value represents the cross point between the x-axis and the tangent crossing the inflection point of the real-time PCR fluorescence curve. For example, in Figure 4, three real-time PCR quantifications starting from the same amount of DNA but in the presence of decreasing amplification mix are shown. In these amplification conditions, the *Ct *method clearly underestimated the samples due to the shift towards the right of *Ct *(Fig. 4A). On the contrary, using the *Cy*_*0 *_methods this shift was clearly correct. In fact, in the presence of PCR inhibition, the fluorescence values of curve inflection points decreased as did the slope of the curve in that point. This resulted in a very small variation of *Cy*_*0 *_values (CV% = 0.6%; Fig. 4B), while the same fluorescence data analysed by *Ct *methods produced a CV% of 1.45% (Fig. 4A).

### Precision and accuracy of the *Ct*, *Cp *and *Cy*_*0 *_methods

**Figure 5:**
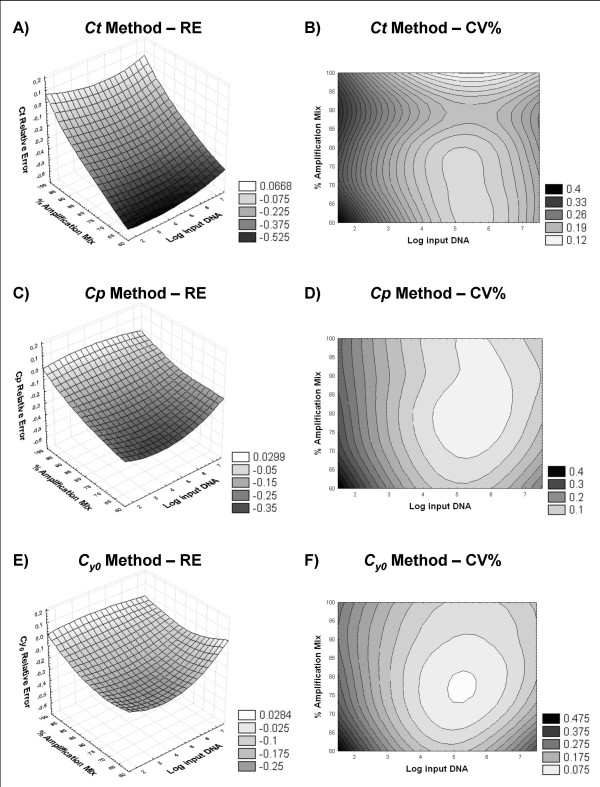
**Comparison of the *Ct*, *Cp *and *Cy*_*0 *_methods in terms of precision and accuracy**. The accuracy of each method has been reported as Relative Error (RE = expected value – estimated value) while the precision was evaluated measuring the variation coefficient (CV%). The 3D plots show the variation of relative error in relation to amplification mix percentage and log_10 _input DNA for the *Ct *(A), *Cp *(C) and *Cy*_*0 *_(E) methods. The areas in the level curve graphs represent the CV% values obtained for each amplification mix percentage and Log_10 _input DNA combination using the *Ct *(B), *Cp *(D) and *Cy*_*0 *_(F) methods.

The performance of the *Ct*, *Cp *and *Cy*_*0 *_methods was compared in terms of precision and accuracy over a wide range of input DNA concentrations and under different reaction efficiencies obtained by decreasing the amount of amplification mix as reported in Liu and Saint [18,27]. As shown in Figure 5A, the *Ct *method is highly rigorous at maximum reaction efficiency regardless of the starting DNA template. However, the absolute value of RE increased almost linearly with the decrease of efficiency regardless of the template concentrations resulting in an underestimation of the unknown of about 50% at the lowest amplification efficiencies. The *Cp *was more accurate than the *Ct *method in the presence of different amounts of amplification mix. Indeed, the relative error in the presence of 100% amplification mix tended towards zero as it did using the *Ct *method. However, when the efficiency declined, the RE increased initially in the same manner at low and high input DNA concentrations, while at 60–70% of the amplification mix, this method markedly underestimated at low concentrations (mean RE_60% mix; _= -0.58; Fig. 5C). Finally, the *Cy*_*0 *_method was more accurate than the *Cp *method (mean RE -0.12 versus -0.18, respectively; Fig. 5C, E), which in turn was better than the *Ct *method (mean RE = -0.31). Notably, at optimal amplification conditions (90–100% of the amplification mix) the *Cp *and *Cy*_*0 *_methods were equivalent, but at decreasing efficiencies, the *Cy*_*0 *_accuracy was more stable than that of the *Cp *in the concentration range from 3.14 × 10^7 ^to 3.14 × 10^5 ^molecules. At lower DNA concentrations, from 3.14 × 10^4 ^to 3.14 × 10^2 ^molecules, the RE proportionally increased with the efficiency decline, but this underestimate was less marked than that of the *Cp *method at the same starting DNA (Fig. 5C, E). Regarding the precision of the three methods, the variation coefficients were determined for each combination of initial template amount and amplification mix percentage. The random error of quantification achieved by the *Cp *and *Cy*_*0 *_method was similar (mean CV% 21.8% and 22.5%, respectively), while the *Ct *procedure produced an overall CV% of about 39.7% (Tab. 3). When the CV was analysed in relation to PCR efficiency and input DNA, an area of low variation coefficients for the three methods was found between 3.14 × 10^4 ^and 3.14 × 10^7 ^molecules as starting material (Fig. 5B, D, F). With DNA amounts ranging from 3.14 × 10^3 ^to 3.14 × 10^2 ^molecules, the precision progressively decreased in each analysis procedure. These variations were not efficiency-dependent, but were related to initial DNA quantity as shown by the shapes of level curves reported in Figure 5B, D and 5F, which were perpendicular to the input template amounts.

**Table 3:** Comparison of mean Relative Error and mean Variation Coefficient among the *Ct, Cp, Cy*_*0 *_and *SCF *methods.

	***Ct***	***Cp***	***Cy***_*0*_	***SCF***	***Log***_10_***SCF***
**Mean CV%**	39.70%	21.80%	22.52%	594.74%^a^	66.12%^a^
**Mean RE**	-0.318	-0.184	-0.128	-5.058^a^	-0.205^a^

The reported data were calculated on 420 PCRs except for ^a^) in which the reaction number was 210.

### Experimental system 2: Real-time PCR quantification in the presence of the inhibitor IgG

**Figure 6:**
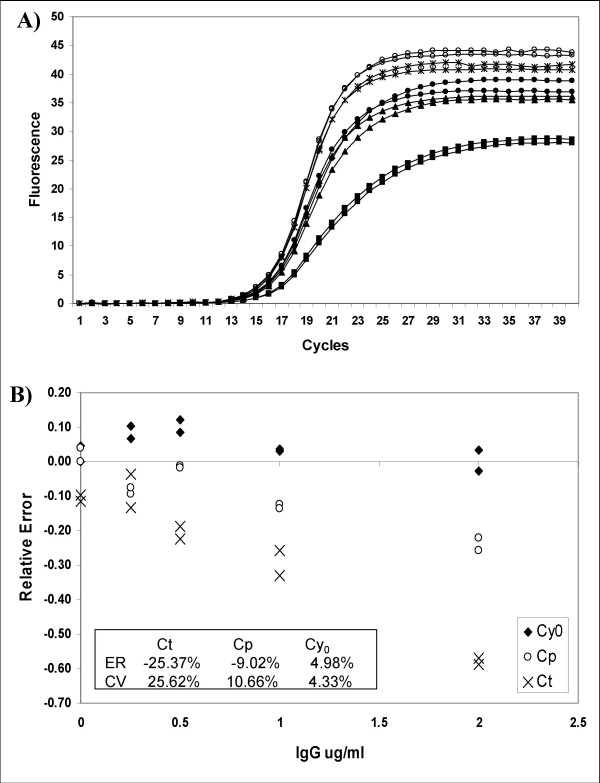
**Real-time PCR amplification plots obtained from the same starting DNA in the presence of IgG acting as reaction inhibitor**. This inhibition system produces curves which are progressively less steep than non-inhibited reactions with increasing IgG concentrations (A). When analysed by the *Ct*, *Cp *and *Cy*_*0 *_methods these curves showed a RE% of -25.37%, -9.02% and 4.98% and a CV% of 25.62%, 10.66% and 4.33%%, respectively (B).

The real-time amplification plot of 4.05 × 10^6 ^DNA molecules with increasing concentrations of IgG demonstrates the effects of PCR inhibition on amplification efficiency and accumulated fluorescence (Fig. 6A). As inhibitor concentrations increased, the amplification curves showed lower plateau fluorescence levels and a shift towards the right and the bottom of the inflection points, leading to amplification curves that were less steep and not as symmetric as those obtained in absence of the inhibitor agent (Fig. 6A). As shown in figure 6A the amplification curves inhibited by IgG showed a shape very similar to those resulting from the system of amplification mix reduction (system 1; Fig. 4A). Quantitative data analysis of these amplification plots showed that the estimated DNA quantities were systematically underestimated in the presence of IgG concentrations higher than 0.25 μg/ml and 1 μg/ml using *Ct *and *Cp *methods, respectively. However, the *Cy*_*0 *_method was able to adjust this bias minimizing the RE at high IgG concentrations (RE = 4.98%; CV = 4.33%; Fig. 6B). Furthermore, in presence of high IgG concentrations, the *SCF *approach, modified according to Rutledge 2004 [26], was inapplicable because it was impossible to minimize *F*_*0 *_value (Additional file 5).

## Discussion

None of the current quantitative PCR data treatment methods is in fact fully assumption-free, and their statistical reliability are often poorly characterized. In this study, we evaluated whether known real-time elaboration methods could estimate the amount of DNA in biological samples with precision and accuracy when reaction efficiencies of the unknown are different from those of the standard curve.

Our experimental systems consisted in the quantification of samples with the same known starting template amount but the amplification reaction, performed for the real-time PCR assay, had a slightly decreasing efficiency. This is clearly not in agreement with the main assumption of the threshold approach which holds that the amplification efficiency of samples has to be identical to, or not significantly different from, that predicted by the standard curve. However, such an assumption has been reported to be patently invalid for many cases in medical diagnostics. In fact, some, if not all, of the biological samples may contain inhibitors that are not present in the standard nucleic acid samples used to construct the calibration curve, leading to an underestimation of the DNA quantities in the unknown samples [28,29]. In our study, slightly decreasing efficiencies were obtained by two systems: diluting the master enzyme mix or adding IgG, a known inhibitor of PCR. Although, the first system is an "in vitro" simulation of PCR inhibition, it produces amplification curves very similar to those obtained in the presence of a biological inhibitor like IgG.

Notably, our experimental setup is not characterized by aberrant amplification reactions. On the contrary, the reactions show a slight mean efficiency decrease which is always the case of biological samples. This PCR inhibition remains undetected when using a threshold approach leading to target underestimation. Moreover, small differences in amplification efficiency produce large quantitative errors and the frequency and magnitude of these errors are virtually impossible to ascertain using a threshold approach. It has been shown that a difference as small as 4% in PCR efficiency could translate into a 400% error in comparative *Ct *method based quantification [24].

Considering previous works [18,19] which demonstrated the capability of the *SCF *method to quantify a sample without prior knowledge of amplification efficiency, our first choice was to process the experimental data by the *SCF *method. The effectiveness of the *SCF *approach is based on curve fitting of raw data so that variations unique to each amplification reaction are incorporated into the analysis. Hence, the results reported herein surprisingly demonstrated that the accuracy and precision of the *SCF *method was markedly impaired when efficiency fell. In fact, when PCR efficiency decreased by about 2.5% (88.8% efficiency value in the presence of 100% of the amplification mix dropped to 84.4% efficiency in the presence of 60% of the mix), we observed, using the *SCF *method with log-transformation, that the RE and CV went from 15% to 43% and from 61% to 55%, respectively.

Furthermore, we found that, when the amplification curve was inhibited, by IgG, the method proposed by Rutledge [26] can not be applied because for each cut-off cycle eliminated from the plateau phase the *F*_*0 *_value progressively decreased without ever reaching a minimum value. These observations are in agreement with two recent studies, which reported that it is possible to obtain absolute quantification from real-time data without a standard curve, but the *Ct *method remains a gold standard due to the inherent errors of the multiple estimates used in nonlinear regression [19,20]. These observations are in accordance with Feller's conclusions that different S-shaped curves can be effectively fitted with various sigmoid models [30], each providing distinct *F*_*0 *_values. Thus sigmoid fit methods such as the logistic model, used in the *SCF *approach, are purely descriptive and quantitative results may be unreliable. This led us to develop a new mathematical data treatment method, named *Cy*_*0*_, based on nonlinear regression fitting of real-time fluorescence data. The proposed method's main advantages are its use of the Richards equation for obtaining the coordinate of the inflection point and the determination of the quantitative entity *Cy*_*0 *_using the five parameters of reaction curve.

Although the logistic growth equation generates a curve that tends towards an exponential form at low fluorescence values, making this curve ideal to model PCR reaction, its maximum slope, or inflection point, is always imposed to be at half the value of the upper asymptote, (*F*_*max*_-*F*_*b*_)/2. This is unsatisfactory because the factors that determine the growth rate are complex and some amplification systems, although characterized by good reaction efficiency, as assessed by standard curve, do not have the center of symmetry in the inflection point. The Richards equation is a more flexible growth function because it has an additional parameter, which is a shape parameter that can make the Richards equation equivalent to the logistic, Gompertz, or monomolecular equations [31,32]. Variation of the shape parameter allows the point of inflection of the curve to be at any value between the minimum and the upper asymptote; when *d *= 1 the Eq. 3 becomes the sigmoidal equation.

Furthermore, since very small errors of the multiple estimates used in non-linear regression lead to large variations in *F*_*0 *_values, the real-time PCR kinetic parameters were used to define a new quantitative entity, the *Cy*_*0*_. The *Cy*_*0 *_relies on the inflection point position and on the slope of the fluorescence curve at that point, so that its value slightly changes in relation to PCR efficiency. In particular, in a slightly inhibited amplification reaction, the fluorescence curves are shifted towards the right and/or they are less steep; this generates higher *Ct *values than those found under optimal amplification conditions, underestimating the target amount. In the *Cy*_*0 *_method, the tangents, calculated from different PCR efficiency, tend to intersect at a common point near the x-axis leading to small variations in the *Cy*_*0 *_values (Fig. 4).

The standard curve approach was chosen for the proposed method because currently there no genuine mathematical model for PCR efficiency assessment. The main complication is that actual efficiency amplification is not constant through the PCR run being high in exponential phase and gradually declining towards the plateau phase [33-35]. However, most current methods of PCR efficiency assessment report "overall" efficiency as a single value [13,24,36,37]. Moreover, recent publications on PCR efficiency assessment have concentrated on the analysis of individual shapes of fluorescence plots in order to estimate a dynamic efficiency value [19,20,27,38]. This proliferation of new methods to assess PCR efficiency demonstrates that, at present, there is not an accepted procedure to evaluate PCR efficiency from a single run, hence some methods can "overestimate" and others "underestimate" the "true" PCR efficiency [8]. In contrast, the standard curve method is based on a simple approximation of data obtained in standard dilutions to unknown samples. In this procedure PCR efficiency assessment is based on the slope of the standard curve. Indeed, the original method (*Ct*) does not account for PCR efficiencies in individual target samples. The proposed procedure overcomes this limitation by evaluating single amplification variations using Richards curve fitting and subsequently produces a *Cy*_*0 *_value that minimizes the dependence of its value on PCR kinetic.

We then compared our method with the *Ct *method, the actual "gold standard" in real-time PCR quantification and the *Cp *method which is also used in molecular diagnostics. Both methods are based on standard curve methodology and are the most frequently used in this field. The *Ct*, *Cp *and *Cy*_*0 *_methods were evaluated on the same data set using two criteria: precision and accuracy. We defined the accuracy of a model as its ability to provide expected concentrations of the known dilutions under different PCR amplification efficiencies. On the contrary, precision is related to the variability of the results obtained from a given model, and it indicates whether reliable results may be obtained from a small data collection. Our results clearly demonstrated that, under optimal amplification conditions, these three methods were equally precise and accurate. However, when the PCR efficiency decreased, due to amplification mix dilution or IgG presence, the *Ct *method was markedly impaired and the *Cp *and *Cy*_*0 *_methods proved to be significantly more accurate than the *Ct *method. Notably, the *Cy*_*0 *_method showed accuracy levels higher than the *Cp *method maintaining the same precision.

The ability to carry out reliable nuclei acid quantification even in sub-optimal amplification conditions is particularly useful when PCR optimization is not possible, as in the case of high-throughput screening of gene expression or biological samples difficult to cleanse of PCR inhibitors.

Furthermore, the *Cy*_*0 *_method is completely objective and assumption-free. Indeed, it does not require the choice of a threshold value and the assumption of similar amplification efficiency between the standard curve and biological samples, necessary in the *Ct *method. Moreover, there is no need to assume that base pair composition and amplicon size do not impact the fluorescence characteristics of SYBR Green I, required in optical calibration methods like *SCF *[19]. Our procedure may have future applications in TaqMan assays, where the Taq DNA polymerase digests a probe labelled with a fluorescent reporter and quencher dye and the signal diverges from the product resulting in non-symmetric amplification curves that can be effectively modelled by Richards equation [39]. Further work is needed to extensively verify the accuracy and precision of the *Cy*_*0 *_method in the presence of other known PCR inhibitors like phenol, haemoglobin, fat and tannic acid [17,22].

## Conclusion

Real-time PCR analysis is becoming increasingly important in biomedical research because of its accuracy, sensitivity and high efficiency. Although, real-time PCR analysis has gained considerable attention, it is far from being a standard assay. The standard methods are quite stable and straightforward but the accuracy of estimates is strongly impaired if efficiency is not equal in all reactions. Furthermore, the assumption of uniform efficiency has been reported to be invalid in many cases regarding medical diagnostics. In fact, the biological samples may contain inhibitors that could lead to different amplification efficiencies among samples.

We propose, in this report, a modified standard curve-based method, called *Cy*_*0*_, that does not require the assumption of uniform reaction efficiency between standards and unknown.

To the best of our knowledge, this is the first method in which the stability and reliability of a standard curve approach is combined with a fitting procedure to overcome the key problem of PCR efficiency determination in real-time PCR nucleic acid quantification. The data reported herein clearly show that the *Cy*_*0 *_method is a valid alternative to the standard method for obtaining reliable and precise nucleic acid quantification even in sub-optimal amplification conditions, such as those found in the presence of biological inhibitors like IgG.

## Abbreviations

Cp: crossing point; Ct: threshold cycle; CV: coefficient of variation; IgG: immunoglobulin G; RE: relative error; SCF: sigmoidal curve fitting.

## Authors' contributions

MG and DS carried out the design of the study, participated in data analysis, developed the *Cy*_*0 *_method and drafted the manuscript. MBLR participated in data collection and analysis and critically revised the manuscript. LS carried out the real-time PCR. VS participated in the design of the study and critically revised the manuscript. All authors read and approved the final manuscript.

## Supplementary Material

Additional file 1 Windows Excel file containing PCR readouts and non-linear fittings. Click here for file

Additional file 2 Windows Word file containing first and second derivative of Richards equation and the mathematical formulas for obtaining the coordinate of the *Cy*_*0 *_point. Click here for file

Additional file 3 Windows Excel file containing the *Ct Cp Cy*_*0 *_*SCF Log*_10 _*SCF *elaborations. Click here for file

Additional file 4 Windows Excel file containing the results obtained with the *SCF *approach based on a previous study by Rutledge 2004. Click here for file

Additional file 5 Windows Excel file containing the results obtained with the *SCF *approach based on a previous study by Rutledge 2004 in presence of IgG. Click here for file
