# Supplementary material for: A new real-time PCR method to overcome significant quantitative inaccuracy due to slight amplification inhibition
Source: BMC Bioinformatics. 2008 Jul 30;9:326. doi: 10.1186/1471-2105-9-326 (PMC2533027; doi:10.1186/1471-2105-9-326)
Supplement: Additional file 2 — Windows Word file containing first and second derivative of Richards equation and the mathematical formulas for obtaining the coordinate of the Cy0 point. [file 1471-2105-9-326-S2.doc]

Additional file 2:

The$C_{y0}$value has been determined as follows:

1) First derivative of Richards equation:

$$\frac{dF_{x}}{dx}=F_{\max}\left(-d\right)\left[1+e^{-\frac{1}{b}\left(x-c\right)}\right]^{-d-1}e^{-\frac{1}{b}\left(x-c\right)}\left(-\frac{1}{b}\right)$$

therefore:

$$\frac{dF_{x}}{dx}=\frac{dF_{\max}}{b}e^{-\frac{1}{b}\left(x-c\right)}\left[1+e^{-\frac{1}{b}\left(x-c\right)}\right]^{-d-1}$$

2) Second derivative of Richards equation:

$$\frac{d^{2}F_{x}}{dx^{2}}=\frac{dF_{\max}}{b}\left\{e^{-\frac{1}{b}\left(x-c\right)}\left(-\frac{1}{b}\right)\left(1+e^{-\frac{1}{b}\left(x-c\right)}\right)^{-d-1}+e^{-\frac{1}{b}\left(x-c\right)}\left(-d-1\right)\left(1+e^{-\frac{1}{b}\left(x-c\right)}\right)^{-d-2}e^{-\frac{1}{b}\left(x-c\right)}\left(-\frac{1}{b}\right)\right\}$$

therefore:

$$\frac{d^{2}F_{x}}{dx^{2}}=-\frac{dF_{\max}}{b^{2}}e^{-\frac{1}{b}\left(x-c\right)}\left[1+e^{-\frac{1}{b}\left(x-c\right)}\right]^{-d-2}\left[1+e^{-\frac{1}{b}\left(x-c\right)}-de^{-\frac{1}{b}\left(x-c\right)}-e^{-\frac{1}{b}\left(x-c\right)}\right]$$

and finally:

$$\frac{d^{2}F_{x}}{dx^{2}}=-\frac{dF_{\max}}{b^{2}}e^{-\frac{1}{b}\left(x-c\right)}\left[1+e^{-\frac{1}{b}\left(x-c\right)}\right]^{-d-2}\left[1-de^{-\frac{1}{b}\left(x-c\right)}\right]$$

3) Determination of abscisse of inflexion point (*x_flex_*):

$\frac{d^{2}F_{x}}{dx^{2}}=0$ when

$$1-de^{-\frac{1}{b}\left(x-c\right)}=0$$

next:

$$x_{flex}=c+b\ln d$$

4) Determination of ordinate in *x_flex_*:

$$Fx_{flex}=\frac{F_{\max}}{\left[1+e^{-\frac{1}{b}\left(c+b\ln d-c\right)}\right]^{d}}+F_{b}$$

therefore:

$$Fx_{flex}=\frac{F_{\max}}{\left[1+e^{\ln d^{-1}}\right]^{d}}+F_{b}$$

$$Fx_{flex}=\frac{F_{\max}}{\left(1+\frac{1}{d}\right)^{d}}+F_{b}$$

and finally:

$$Fx_{flex}=F_{\max}\left(\frac{d}{d+1}\right)^{d}+F_{b}$$

5) Determination of the slope of tangent strait-line (*m*) crossing the inflection point:

*m* =$F^{'}x_{flex}=\frac{dF_{\max}}{b}e^{-\frac{1}{b}\left(c+b\ln d-c\right)}\left[1+e^{-\frac{1}{b}\left(c+b\ln d-c\right)}\right]^{-d-1}$

$$F^{'}x_{flex}=\frac{dF_{\max}}{b}\left(\frac{1}{d}\right)\left[1+\frac{1}{d}\right]^{-d-1}$$

therefore:

*m* =$F^{'}x_{flex}=\frac{F_{\max}}{b}\left[\frac{d}{d+1}\right]^{d+1}$

the tangent straight-line equation in flex point is:

$$y-Fx_{flex}=F^{'}x_{flex}\left(x-x_{flex}\right)$$

intersection point (*Cy_0_*) between abscise axis and tangent straight-line is:

$$C_{y0}=\frac{Fx_{flex}}{F^{'}x_{flex}}+x_{flex}$$

from which:

$$C_{y0}=\frac{F_{\max}\left(\frac{d}{d+1}\right)^{d}+F_{b}}{\frac{F_{\max}}{b}\left[\frac{d}{d+1}\right]^{d+1}}+c+b\ln d$$

next:

$$C_{y0}=c+b\ln d+b\left(\frac{d+1}{d}\right)^{d+1}\left[\left(\frac{d}{d+1}\right)^{d}+\frac{F_{b}}{F_{\max}}\right]$$

and finally:

$$C_{y0}=c+b\ln d-b\left(\frac{d+1}{d}\right)\left[1-\frac{F_{b}}{F_{\max}}\left(\frac{d+1}{d}\right)^{d}\right]$$
